# What Is the Need for and Access to Trauma Surgery in Low‐ and Middle‐Income Countries? A Scoping Review

**DOI:** 10.1002/wjs.12626

**Published:** 2025-05-19

**Authors:** Thomas Edmiston, Michael F. Bath, Amila Ratnayake, Luis Felipe Reyes, Eleanor Reffin, Zhongheng Zhang, Raoof Saleh, Eder Caceres, Joachim Amoako, Isla Kuhn, Brandon G. Smith, Lekaashree Rambabu, Orla Mantle, Vedha Penmetcha, Laura Hobbs, Katharina Kohler, Timothy C. Hardcastle, Thomas G. Weiser, Tom Bashford

**Affiliations:** ^1^ International Health Systems Group Department of Engineering University of Cambridge Cambridge UK; ^2^ Department of Surgery Army Hospital Colombo Sri Lanka; ^3^ Clínica Universidad de la Sabana Chía Cundinamarca Colombia; ^4^ School of Medicine Unisabana Center for Translational Science Universidad de La Sabana Chia Colombia; ^5^ King's College Hospital NHS Foundation Trust London UK; ^6^ Department of Emergency Medicine Sir Run Run Shaw Hospital Zhejiang University School of Medicine Hangzhou China; ^7^ Key Laboratory of Precision Medicine in Diagnosis and Monitoring Research of Zhejiang Province Sir Run Run Shaw Hospital Zhejiang University School of Medicine Hangzhou China; ^8^ School of Medicine Shaoxing University Shaoxing China; ^9^ Longquan Industrial Innovation Research Institute Lishui China; ^10^ Médicins Sans Frontières Medical Unit Berlin Germany; ^11^ University of Ghana Medical School Accra Ghana; ^12^ University of Cambridge Medical Library University of Cambridge Cambridge UK; ^13^ NIHR Global Health Research Group on Acquired Brain and Spine Injury University of Cambridge Cambridge UK; ^14^ Department of Medicine University of Cambridge Cambridge UK; ^15^ KwaZulu‐Natal Department of Health Trauma and Burns Unit Inkosi Albert Luthuli Central Hospital Durban South Africa; ^16^ Department of Surgical Sciences Nelson R Mandela School of Clinical Medicine University of KwaZulu‐Natal Durban South Africa; ^17^ Department of Surgery Stanford University Stanford California USA; ^18^ Department of Anaesthesia Cambridge University Hospitals NHS Foundation Trust Cambridge UK

**Keywords:** global health, surgery, trauma

## Abstract

**Introduction:**

Trauma is a major source of morbidity and mortality globally, but low‐ and middle‐income countries (LMICs) are disproportionately affected by higher volumes of trauma and worse health outcomes. Despite this, there are limited data describing how many individuals in these regions need trauma surgery, and how many are able to access it.

**Methods:**

We performed a scoping review to examine the current available evidence on the need for, and access to, trauma surgery in LMICs in accordance with the Preferred Reporting Items for Systematic Reviews and Meta‐Analyses extension for Scoping Reviews guidelines. We included studies published after 2000, across all languages, that reported data on LMICs, as defined by the Organization for Economic Cooperation and Development.

**Results:**

We identified 32 articles describing the need for trauma surgery and 24 articles describing the access to trauma surgery, representing 27 LMICs overall. The median rate of trauma need was 7361 individuals per 100,000 per year (IQR 6313–9461), whereas the median rate of trauma surgery was 64.8 procedures per 100,000 per year.

**Conclusion:**

Our study suggests that the need for trauma surgery is far greater than the access provided in LMICs. Indeed, the median rate of trauma surgery currently performed in the represented LMICs was 20 times less than the Lancet Commission on Global Surgery's benchmark. This scoping review illustrates the pressing requirement to generate high‐quality prospective data to describe trauma care in LMICs.

## Graphical abstract

Trauma is a major source of morbidity and mortality globally, but low‐ and middle‐income countries (LMICs) are disproportionately affected by higher volumes of trauma and worse health outcomes. Despite this, there are limited data describing how many individuals in these regions need trauma surgery, and how many are able to access it. We performed a scoping review to examine the current available evidence on the need for, and access to, trauma surgery in LMICs in accordance with the Preferred Reporting Items for Systematic Reviews and Meta‐Analyses extension for Scoping Reviews guidelines.

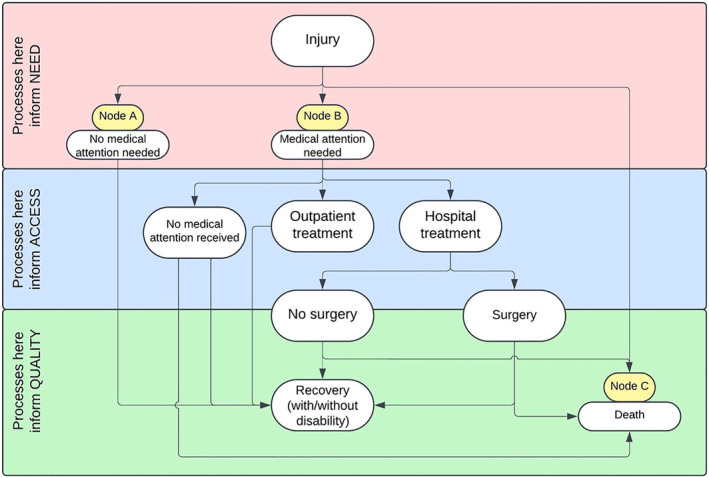

## 1 Introduction

Trauma is a major cause of death and disability worldwide. The 2019 Global Burden of Disease study found that around 8% of all deaths and nearly one in 10 of all lost disability‐adjusted life years (DALYs) was associated with traumatic injuries [1]. Moreover, it has been estimated that around 90% of deaths from trauma occur in low‐ and middle‐income countries (LMICs) [2], and that two million lives could be saved annually if LMICs achieved the same outcomes as high‐income countries (HICs) [3]. Trauma systems have been implemented across several high‐income countries over the last few decades to provide integrated care [4, 5, 6, 7] and have been shown to significantly improve patient outcomes [8, 9]; however, the majority of these systems implemented have been within high‐income countries (HICs), the designs of which are often nontransferable to the LMIC setting [10, 11]. Indeed, any trauma system development in the LMIC setting requires high quality and context‐specific data available, yet there is a paucity of data on current trauma care in LMICs, especially in the surgical procedures performed—a fundamental component of trauma care.

**FIGURE 1:**
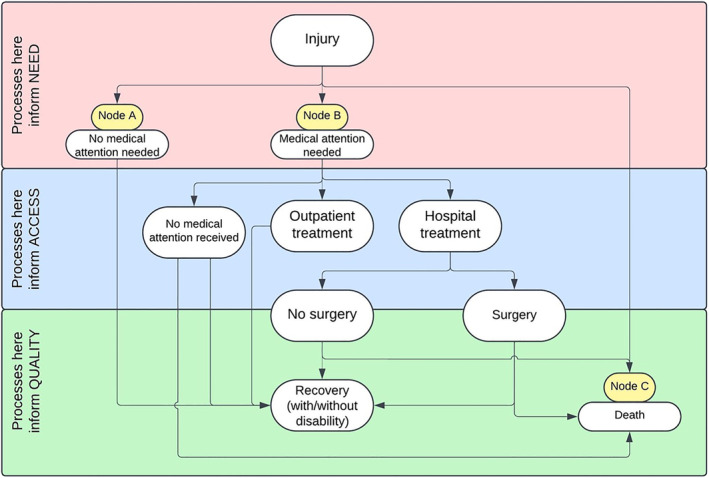
A schematic of the potential pathways of a trauma patient and how they relate to access, need, and quality.

A framework of need, access, and quality has been used to describe the remit of global surgical care [12]. In this context, need represents the population requiring a surgical intervention, access represents the subgroup of patients who are able to receive it, and quality represents the safety and effectiveness of this intervention. The potential healthcare journey a patient takes following an injury is hugely variable and influenced by factors across need, access, and quality (Figure 1). Understanding the need for trauma surgery is particularly challenging, as injuries happen in the community and many patients never reach formalized medical care, making effective data collection challenging. The Nodes A, B, and C in Figure 1 refer to patient groups that are commonly described in studies that examine rates of trauma in the community and hence can be used to infer the need for trauma surgery; however, it is important to note that none solely is the representative of the “true” need for trauma surgery.

If trauma care and trauma systems are to be improved globally, understanding the current need for trauma surgery, and the access currently provided, is paramount. In this study, we aim to characterize the need for, and access to, trauma surgery in LMICs globally as described in the existing literature.

## 2 Methods

This study was conducted in accordance with the Preferred Reporting Items for Systematic Reviews and Meta‐Analyses extension for Scoping Reviews (PRISMA‐ScR) framework [13]. The review protocol was published in an open‐access peer‐reviewed journal [14]. No patients or public were involved in the design of the study. Following a preliminary review of the literature, given the paucity and heterogeneity of data apparent on trauma surgery in LMICs, a scoping review was deemed most appropriate to present the current evidence on the topic.

### 2.1 Eligibility Criteria

Trauma was defined as any “significant injury or injuries that have potential to be life‐threatening or life‐changing sustained from either high energy mechanisms or low energy mechanisms in those rendered vulnerable by extremes of age” [15]. Therefore, trauma surgery was defined as any acute surgery necessary to provide treatment for such trauma. Studies across neurosurgery, thoracic, abdominal, or orthopedic (including amputations) surgery were included. Given the presumed limited and heterogeneous data available on this topic, it was decided a priori to focus on surgical procedures as an indicator of access, as opposed to including wider trauma management services (e.g., intensive care services and nonoperative management), aiming to provide a more quantifiable and directly comparable data point.

A low‐ and middle‐income country (LMIC) was defined as any country on the Development Assistance Committee (DAC) list of Official Development Assistance (ODA) recipients as provided on the website of the Organization for Economic Cooperation and Development (OECD) [16]. We included any observational study conducted in LMICs on a multicenter, national, or multinational scale, with both adult and pediatric studies included. Studies in all languages were included, with only studies published after 1st January 2000 included, to ensure outdated data did not bias our findings.

All nonobservational or interventional studies were excluded. Single‐center studies were also excluded, as these studies often draw from small populations with high variance in need and access to trauma surgery, therefore may have biased the study findings. To ensure local healthcare services only were represented, studies where care was provided by foreign providers (e.g., nongovernmental organizations or international charities) or studies in the setting of natural disasters and military healthcare were excluded. To ensure appropriate population‐level trauma data were collected, we further excluded studies describing trauma to a specific organ (e.g., splenic injury), trauma by a specific mechanism (e.g., road traffic accidents), or trauma affecting a particular subgroup of patients (e.g., intensive care patients). For multinational studies that included both HICs and LMICs, we extracted the data for LMIC patients only and where this was not possible the study was excluded.

### 2.2 Search Strategy

We devised a literature search strategy in collaboration with a medical librarian (IK) and conducted searches of MEDLNE via Ovid, Global Health via EBSCO, Web of Science, and Global Index Medicus (a full search strategy can be found in the Supporting Information S1: Appendix). Grey literature and referenced studies were also included where appropriate.

**FIGURE 2:**
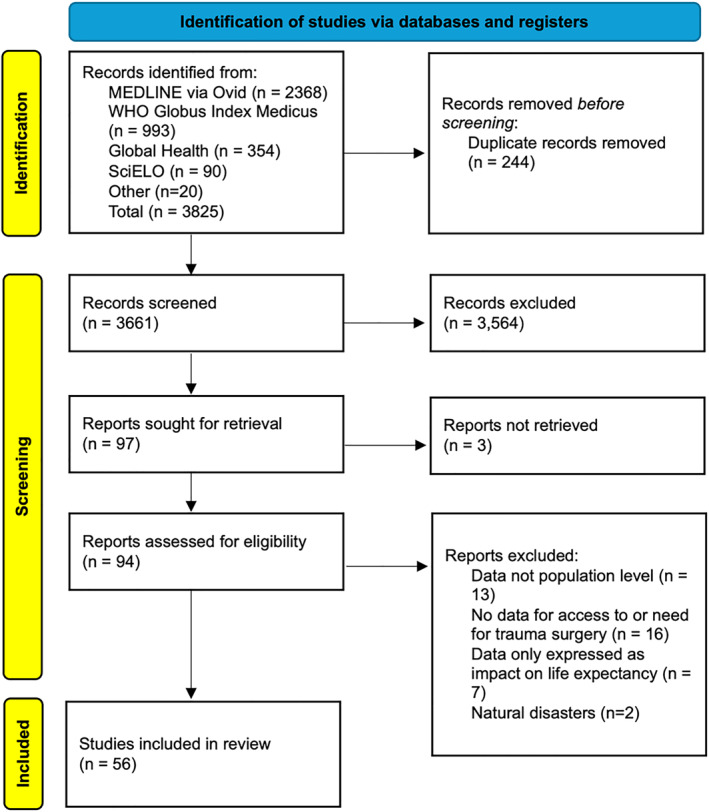
PRISMA flowchart.

Articles identified through our search strategy were uploaded to Rayyan [17] and two authors (TE and ER) independently screened their titles and abstracts for inclusion. Any disagreements were resolved by a third author (MB). The full body of articles meeting the inclusion criteria were then assessed for inclusion in the scoping review [18] (Figure 2). References of included articles were also screened to source further studies for inclusion.

### 2.3 Data Collection

The study design, the location of the study, and the type of trauma (either all‐type or subtype) were recorded. Where possible, the most frequent mechanisms of trauma in the study cohort were recorded.

For studies describing trauma surgery need, data were standardized into trauma surgeries required per 100,000 people per year. For studies describing access to trauma surgery, data were standardized into trauma surgeries performed per 100,000 per year; if no denominator representing the population served was available, the unadjusted number of surgical procedures performed per year was reported.

### 2.4 Data Analysis

Given the heterogeneity of the included studies and variable data quality, only descriptive statistics were performed.

All included studies were assessed for risk of bias, using the Critical Appraisal Skills Program (CASP) for descriptive/cross‐sectional studies tool [19]. For non‐English studies, appraisal was performed by a native‐language speaker.

## 3 Results

Through the initial literature search, 3825 articles were identified, including 20 from grey literature and reference searching. Following removal of duplicates and screening, 56 were included in the final review (Figure 2). Of these, 32 described the need for trauma surgery [20, 21, 22, 23, 24, 25, 26, 27, 28, 29, 30, 31, 32, 33, 34, 35, 36, 37, 38, 39, 40, 41, 42, 43, 44, 45, 46, 47, 48, 49, 50, 51] and 24 described access to trauma surgery [52, 53, 54, 55, 56, 57, 58, 59, 60, 61, 62, 63, 64, 65, 66, 67, 68, 69, 70, 71, 72, 73, 74, 75].

**FIGURE 3:**
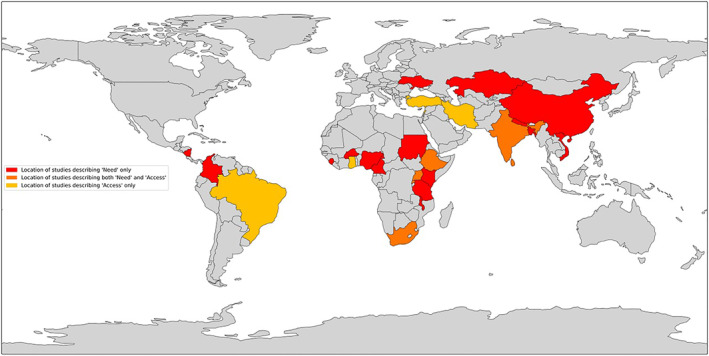
Map showing the location of the included studies.

Twenty seven countries were represented by the 56 studies included (Figure 3), which constitutes approximately 20% of all LMICs globally [76]. Data regarding the need for trauma surgery only, the access to trauma surgery only, and both need and access were present in 5, 16, and 6 countries, respectively.

### 3.1 Need

Of the 32 studies identified describing the need for trauma surgery, 30 described all trauma and two described orthopedic trauma only (Table 1). All studies were retrospective in nature, with the majority (*n* = 23) utilizing population‐based surveys, and the median study population was 9568 patients (IQR 3645–115,000); one study where population was provided as “households” was excluded from this calculation. Fourteen studies surveyed victims of injury (Figure 1 Nodes A and B), six studies surveyed those injured that needed medical attention only (Figure 1 Node B), and 12 studies surveyed trauma‐related mortality only (Figure 1 Node C). The majority of studies were assessed as having either a low or medium risk of bias.

**TABLE 1:** Included studies describing the need for trauma surgery.

Authors	Year	Study design	Study scale	Country	Type of trauma	Node(s)	Study population size	Trauma surgery need, per 100,000 per year	Risk of bias
Whitaker et al. [20]	2024	Cross‐sectional population‐based survey	National	Malawi	All	A,B	2200^b^	6900	Medium
Whitaker et al. [21]	2021	Cross‐sectional population‐based survey	Regional	Burkina Faso	All	A, B	3028	7662	Medium
Christie et al. [22]	2020	Cross‐sectional population‐based survey	National	Cameroon	All	A, B	8046	5854	Medium
Petroze et al. [23]	2015	Cross‐sectional population‐based survey	National	Rwanda	All	A, B	3175	7060	Medium
Stewart et al. [24]	2013	Cross‐sectional population‐based survey	National	Sierra Leone	All	A, B	3645	12,401	Medium
El Tayeb et al. [25]	2013	Cross‐sectional population‐based survey	Regional	Sudan	All	A, B	5561	7790	Medium
Navaratne et al. [26]	2009	Cross‐sectional population‐based survey	Regional	Sri Lanka	All	A, B	9568	24,600	Medium
Atijosan et al. [27]	2009	Cross‐sectional population‐based survey	National	Rwanda	Orthopedic	A, B	3526	—	Low
Ma et al. [28]	2008	Cross‐sectional population‐based survey	Regional	China	All	A, B	24,438	6772	Medium
Atijosan et al. [29]	2008	Cross‐sectional population‐based survey	National	Rwanda	Orthopedic	A, B	8367	—	Low
Olawale et al. [30]	2007	Cross‐sectional population‐based survey	Regional	Nigeria	All	A, B	1766	10,022	Medium
Tercero et al. [31]	2006	Cross‐sectional population‐based survey	Regional	Nicaragua	All	A, B	63,886	4142	Medium
Moshiro et al. [32]	2005	Cross‐sectional population‐based survey	Regional	Tanzania	All	A, B	15,223	3270	Medium
Hang et al. [33]	2005	Cross‐sectional population‐based survey	Regional	Vietnam	All	A, B^a^	24,776	8900	Medium
Gathecha et al. [34]	2018	Cross‐sectional population‐based survey	National	Kenya	All	B	4484	15,165	Medium
Varela et al. [35]	2017	Cross‐sectional population‐based survey	National	Malawi	All	B	2909	6085	Medium
Tran et al. [36]	2017	Cross‐sectional population‐based survey	National	Uganda	All	B	4248	1083	Medium
Butler et al. [37]	2016	Cross‐sectional population‐based survey	National	Uganda	All	B	2176	—	Medium
Gupta et al. [38]	2015	Cross‐sectional population‐based survey	National	Nepal	All	B	2695	—	Medium
Hardcastle et al. [51]	2013	Cross‐sectional study	Regional	South Africa	All	B	11,000,000	1160	Medium
Kosherbayeva et al. [39]	2024	Cross‐sectional study	National	Kazakhstan	All	C	19,000,000	56.7	Low
Mekonnen et al. [40]	2024	Cross‐sectional study, via verbal autopsy	Regional	Ethiopia	All	C	40,895	83.4	Low
Zergaw et al. [41]	2023	Cross‐sectional study	National	Ethiopia	All	C	26,700,000	16	Low
Sil et al. [42]	2021	Cross‐sectional study	National	India	All	C	1,756,867	141	Low
Fenta et al. [43]	2021	Cross‐sectional study, via verbal autopsy	Regional	Ethiopia	All	C	7911	37.7	Low
Edem et al. [44]	2019	Cross‐sectional study, via verbal autopsy	Regional	South Africa	All	C	115,000	54.8	Medium
Segura‐Cardona et al. [45]	2018	Cross‐sectional study	National	Colombia	All	C	641,837	81.7	Low
Lekhan et al. [46]	2015	Cross‐sectional study	National	Ukraine	All	C	45,500,000	95	Low
Eddleston et al. [47]	2007	Cross‐sectional study	Regional	Sri Lanka	All	C	3656	48.5	High
Ali et al. [48]	2007	Cross‐sectional study	Regional	China	All	C	124,204	49.9	Low
Hadi [49]	2005	Cross‐sectional study	National	Bangladesh	All	C	62,000	28.3	Low
Moshiro et al. [50]	2001	Retrospective cross‐sectional population‐based survey	Regional	Tanzania	All	C	25,541	78.4	Medium

^a^ Excluded intentional injuries.

^b^ Households only.

A denominator was able to be identified in 27 studies to calculate trauma need as a rate per 100,000 people per year. For studies that surveyed victims of injury (both nodes A and B, *n* = 12), the median need for trauma surgery was 7361 individuals per 100,000 per year (IQR 6313–9461). For studies that surveyed victims of injury requiring medical attention (node B only, *n* = 4), the median need was 3622.5 individuals per 100,000 per year (IQR 1121.5–10625). For studies that surveyed trauma‐related mortality only (node C, *n* = 12), the median need was 55.8 individuals per 100,000 per year (IQR 43.1–82.55).

Falls and road traffic collisions (RTCs) were the most commonly cited mechanism of trauma. Only 4 studies reported the barriers to seeking healthcare, which included belief that the injury was not serious enough (*n* = 3), difficulties with finding transport (*n* = 3), financial costs (*n* = 2), and a lack of trust in local health facilities (*n* = 2).

### 3.2 Access

Of the 24 studies identified describing access to trauma surgery, 11 described all forms of trauma surgery, four focused on abdominal trauma surgery, four focused on amputations, three focused on neurosurgical procedures, and two focused on thoracic trauma surgery (Table 2). The majority of studies were retrospective in nature and were multicenter studies. The majority of studies were assessed as having either a low or medium risk of bias.

**TABLE 2:** Included studies describing the access to trauma surgery.

Authors	Year	Study design	Study scale	Country	Type of trauma	Number of procedures/year	Number of trauma surgeries per 100,000 per year	Risk of bias
Ajiko et al. [52]	2021	Retrospective cross‐sectional study	National	Uganda	All	605	3.37	Medium
Grabski et al. [53]	2021	Prospective observational study	Multicenter	Uganda	All	129	—	Medium
Gyedu et al. [54]	2021	Retrospective cross‐sectional study	National	Ghana	All	6302	59.6	Medium
Gyedu et al. [55]	2020	Retrospective cross‐sectional study	National	Ghana	All	20,183	75.6	Medium
Starr et al. [56]	2019	Prospective cross‐sectional study	Multicenter	Ethiopia	All	492	171	Medium
Shivasabesan et al. [57]	2019	Retrospective review of prospectively collected data	Multicenter	India	All	1938	—	Low
Mansourati et al. [58]	2018	Prospective observational study	Multicenter	India	All	1651	—	Low
Bradshaw et al. [59]	2017	Prospective cross‐sectional study	Multinational	—	All	2844	—	High
Roy et al. [60]	2016	Prospective cross‐sectional study	Multicenter	India	All	1567	—	Low
Shaikh et al. [61]	2015	Retrospective cross‐sectional study	Multicenter	India	All	45,720	69.9	Low
Walker et al. [62]	2010	Retrospective cross‐sectional study	Multicenter	Uganda	All	1619	54	Low
Twahirwa et al. [63]	2021	Retrospective cross‐sectional study	Multicenter	Rwanda	Abdominal	40	—	Medium
Chu et al. [64]	2021	Retrospective cross‐sectional study	Multicenter	South Africa	Abdominal	325	—	Medium
Ehlers et al. [65]	2021	Retrospective cross‐sectional study	Multicenter	South Africa	Abdominal	1850	—	Low
Spence et al. [66]	2016	Prospective cohort study	Multicenter	South Africa	Abdominal	524	22.9	Low
Yaghi et al. [67]	2012	Prospective cross‐sectional study	National	Lebanon	Amputation	79	0.02	Medium
Mousavi et al. [68]	2012	Retrospective cross‐sectional study	Multicenter	Iran	Amputation	30	—	High
Moini et al. [69]	2009	Retrospective cross‐sectional study	National	Iran	Amputation	41	—	Medium
Dogan et al. [70]	2008	Retrospective cross‐sectional study	Multicenter	Turkey	Amputation	19	—	High
Koester et al. [71]	2023	Prospective survey	National	Brazil	Neurosurgical	37,514	17.3	High
Laeke et al. [72]	2021	Prospective cross‐sectional study	Multicenter	Ethiopia	Neurosurgical	264	—	Low
Harrichandparsad et al. [73]	2019	Prospective cross‐sectional study	Multicenter	South Africa	Neurosurgical	1020	—	Medium
Mathangasinghe et al. [74]	2020	Prospective cross‐sectional study	Multicenter	Sri Lanka	Thoracic	324	—	Medium
Ramirez et al. [75]	2018	Retrospective cross‐sectional study	Multicenter	Rwanda	Thoracic	2	—	Medium

For all‐cause trauma, a denominator population to calculate the trauma access as a rate per 100,000 people per year was able to be identified in six studies. The median rate of trauma surgery was 64.8 per 100,000 per year (IQR 54–75.6). Five studies covering all types of trauma surgery provided a breakdown of procedures by subspecialties, of which orthopedic procedures were the most frequently performed.

Eight studies provided a breakdown of mechanisms of trauma leading to surgery. RTCs were the most common mechanism of trauma patients having surgery (mean of 50.6% across studies reporting mechanisms), followed by falls (28.8%) and assault or interpersonal violence (11.4%).

## 4 Discussion

We have demonstrated that there are sizable differences between the number of traumatic injuries sustained and the number of procedures currently performed for trauma in LMICs globally. Moreover, the current levels of access to trauma surgery undertaken in LMICs is far below that reported in many HICs. Previous work has demonstrated that in excess of two million lives could be saved in LMICs if the same standards of care from HICs were implemented [3], however if global trauma care outcomes are to be improved, then addressing the deficits between need and access in LMICs is paramount.

This scoping review identified 32 studies describing the need for trauma surgery and 24 studies that described levels of access to trauma surgery in LMICs. From these studies, we were able to calculate estimates for the median rate of injury at 7361 patients per 100,000 per year, whereas the calculated median rate for trauma surgeries was 65 operations per 100,000 per year. Although all those injured may not necessarily require medical intervention, these data does suggest a vast disparity regardless between those suffering traumatic injuries and those actually undergoing a surgical procedure. New Zealand has been previously used as a benchmark for the relationship between surgical disease prevalence and utilization of surgical services, as its healthcare system is assumed to be able to access surgical care to its entire population when needed [77]. Their reported rate of trauma surgery is approximately 1158 procedures per 100,000 individuals per year [78]; therefore, based on the data from this scoping reviews, LMICs are currently performing trauma surgery at a rate that is 5.6% of New Zealand's. This means that current access to trauma surgery in LMICs is around 20 times less than this Lancet Commission on Global Surgery's benchmark.

Quantifying the need for trauma surgery is challenging. For example, although higher incidences of injury have often been reported in rural settings compared to urban areas [26, 28, 32], obtaining accurate estimates in such regions can prove challenging due to limited public health surveillance and variable transport links. Previous studies in Sub‐Saharan Africa have shown that rural populations suffer from limited access to trauma care, particularly in less urbanized countries [79, 80, 81, 82]. Furthermore, geographical analyses used to model accessibility often fail to account for socioeconomic and cultural barriers in accessing trauma care [83]. In this review, population‐based surveys were utilized by many included studies, with participants asked to self‐report historic injuries retrospectively, however such methodologies often lead to overestimation of the injury burden. In comparison, an alternative common method used is through inspection of local or national mortuary data to identify deaths due to injury, yet this method often leads to significant underestimation of trauma need. Community‐based prospective studies in both rural and urban environments are key to truly understanding current trauma care standards; such research would be most effective if standardized so that data could be easily cross‐referenced and assimilated, using existing tools, such as the WHO standardized clinical form and new data templates, generated for this specific research [84].

The reasons for the demonstrated disparity between trauma surgery need and access in this study are likely to be context‐dependent and influenced by local factors. However, it is important to note that the populations represented by the included studies are heterogeneous and diverse, as are the healthcare systems they access [2], and caution is advised in treating these data as universal across all LMIC settings. Although we did identify common barriers in receiving trauma care, such as access to road transport or financial costs, detailed research is required in local contexts to identify barriers in individual trauma systems.

We attempted to address a number of limitations with the identified studies in our review. Although we utilized a standardized definition of trauma and trauma surgery, there remained significant heterogeneity in study design and study populations [15]. Moreover, often traumatic injuries were classified with other causes, such as burns, poisoning, drowning, or frostbite, as described in the ICD classification [85], which may lead to an overestimation of injuries. Finally, there was minimal direct overlap between the populations described by the studies identifying need and access, respectively, with only one study on a national scale describing both need and access, limiting the validity of the conclusions that can be drawn.

## 5 Conclusion

This scoping review demonstrates that the need for trauma surgery in LMICs far outstrips current levels of access, with the rates of trauma surgery well below the Lancet Commission on Global Surgery's benchmark metric. The relationship between these two facets of trauma patient care is complex and reflects the requirement for further investment in this field of research if improvements to global trauma care are to be made.

## Author Contributions

**Thomas Edmiston:** conceptualization, methodology, formal analysis, writing – original draft, writing – review and editing, data curation. **Michael F. Bath:** conceptualization, methodology, supervision, writing – original draft, writing – review and editing, data curation, investigation, formal analysis. **Amila Ratnayake:** methodology, formal analysis, writing – review and editing, writing – original draft. **Luis Felipe Reyes:** methodology, formal analysis, writing – review & editing, writing – original draft. **Eleanor Reffin:** data curation, formal analysis. **Zhongheng Zhang**: methodology, writing – review and editing, writing – original draft. **Raoof Saleh:** methodology, writing – review and editing, writing – original draft. **Eder Caceres:** methodology, writing – review and editing, writing – original draft. **Joachim Amoako:** conceptualization, methodology, writing – review and editing, writing – original draft. **Isla Kuhn:** data curation, methodology. **Brandon G. Smith:** methodology, conceptualization. **Lekaashree Rambabu:** methodology, writing – original draft. **Orla Mantle:** methodology, writing – original draft. **Vedha Penmetcha:** methodology, writing – original draft. **Laura Hobbs:** methodology, writing – original draft, writing – review and editing, conceptualization. **Katharina Kohler:** methodology, writing – original draft, writing – review and editing, conceptualization. **Timothy C. Hardcastle:** methodology, conceptualization, investigation, supervision, writing – review and editing. **Thomas G. Weiser:** conceptualization, methodology, investigation, supervision, writing – review and editing. **Tom Bashford:** conceptualization, methodology, writing – review and editing, investigation.

## Ethics Statement

The authors have nothing to report.

## Conflicts of Interest

The authors declare no conflicts of interest.

## Supporting information

Supporting Information S1

## Data Availability

The authors have nothing to report.
