# Supplementary material for: What Is the Need for and Access to Trauma Surgery in Low‐ and Middle‐Income Countries? A Scoping Review
Source: World J Surg. 2025 May 19;49(7):1928–40. doi: 10.1002/wjs.12626 (PMC12282569; doi:10.1002/wjs.12626)
Supplement: Supplementary file 1 — Supporting Information S1 [file WJS-49-1928-s001.docx]

# Appendix

**Ovid Medline Search Strategy:**

Ovid MEDLINE(R) and Epub Ahead of Print, In-Process, In-Data-Review & Other Non-Indexed Citations, Daily and Versions <1946 to May 08, 2024>

1 afghanistan/ or africa/ or africa, northern/ or africa, central/ or africa, eastern/ or "africa south of the sahara"/ or africa, southern/ or africa, western/ or albania/ or algeria/ or andorra/ or angola/ or "antigua and barbuda"/ or argentina/ or armenia/ or azerbaijan/ or bahamas/ or bahrain/ or bangladesh/ or barbados/ or belize/ or benin/ or bhutan/ or bolivia/ or borneo/ or "bosnia and herzegovina"/ or botswana/ or brazil/ or brunei/ or bulgaria/ or burkina faso/ or burundi/ or cabo verde/ or cambodia/ or cameroon/ or central african republic/ or chad/ or exp china/ or comoros/ or congo/ or cote d'ivoire/ or croatia/ or cuba/ or "democratic republic of the congo"/ or cyprus/ or djibouti/ or dominica/ or dominican republic/ or ecuador/ or egypt/ or el salvador/ or equatorial guinea/ or eritrea/ or eswatini/ or ethiopia/ or fiji/ or gabon/ or gambia/ or "georgia (republic)"/ or ghana/ or grenada/ or guatemala/ or guinea/ or guinea- bissau/ or guyana/ or haiti/ or honduras/ or independent state of samoa/ or exp india/ or indian ocean islands/ or indochina/ or indonesia/ or iran/ or iraq/ or jamaica/ or jordan/ or kazakhstan/ or kenya/ or kosovo/ or kuwait/ or kyrgyzstan/ or laos/ or lebanon/ or liechtenstein/ or lesotho/ or liberia/ or libya/ or madagascar/ or malaysia/ or malawi/ or mali/ or malta/ or mauritania/ or mauritius/ or mekong valley/ or melanesia/ or micronesia/ or monaco/ or mongolia/ or montenegro/ or morocco/ or mozambique/ or myanmar/ or namibia/ or nepal/ or nicaragua/ or niger/ or nigeria/ or oman/ or pakistan/ or palau/ or exp panama/ or papua new guinea/ or paraguay/ or peru/ or philippines/ or qatar/ or "republic of belarus"/ or "republic of north macedonia"/ or romania/ or exp russia/ or rwanda/ or "saint kitts and nevis"/ or saint lucia/ or "saint vincent and the grenadines"/ or "sao tome and principe"/ or saudi arabia/ or serbia/ or sierra leone/ or senegal/ or seychelles/ or singapore/ or somalia/ or south africa/ or south sudan/ or sri lanka/ or sudan/ or suriname/ or syria/ or taiwan/ or tajikistan/ or tanzania/ or thailand/ or timor-leste/ or togo/ or tonga/ or "trinidad and tobago"/ or tunisia/ or turkmenistan/ or uganda/ or ukraine/ or united arab emirates/ or uruguay/ or uzbekistan/ or vanuatu/ or venezuela/ or vietnam/ or west indies/ or yemen/ or zambia/ or zimbabwe/ 1340741

2 "Organisation for Economic Co-Operation and Development"/ 602

3 australasia/ or exp australia/ or austria/ or baltic states/ or belgium/ or exp canada/ or chile/ or colombia/ or costa rica/ or czech republic/ or exp denmark/ or estonia/ or europe/ or finland/ or exp france/ or exp germany/ or greece/ or hungary/ or iceland/ or ireland/ or israel/ or exp italy/ or exp japan/ or korea/ or latvia/ or lithuania/ or luxembourg/ or mexico/ or netherlands/ or new zealand/ or north america/ or exp norway/ or poland/ or portugal/ or exp "republic of korea"/ or "scandinavian and nordic countries"/ or slovakia/ or slovenia/ or spain/ or sweden/ or switzerland/ or turkey/ or exp united kingdom/ or exp united states/ 3549964

4 European Union/ 17992

5 Developed Countries/ 21540

6 or/2-5 3566284

7 1 not 6 1249933

8 (afghanistan or albania or algeria or american samoa or angola or "antigua and barbuda" or antigua or barbuda or argentina or armenia or armenian or aruba or azerbaijan or bahrain or bangladesh or barbados or republic of belarus or belarus or byelarus or belorussia or byelorussian or belize or british honduras or benin or dahomey or bhutan or bolivia or "bosnia and herzegovina" or bosnia or herzegovina or botswana or bechuanaland or brazil or brasil or bulgaria or burkina faso or burkina fasso or upper volta or burundi or urundi or cabo verde or cape verde or cambodia or kampuchea or khmer republic or cameroon or cameron or cameroun or central african republic or ubangi shari or chad or chile or china or colombia or comoros or comoro islands or iles comores or mayotte or democratic republic of the congo or democratic republic congo or congo or zaire or costa rica or "cote d'ivoire" or "cote d' ivoire" or cote divoire or cote d ivoire or ivory coast or croatia or cuba or cyprus or czech republic or czechoslovakia or djibouti or french somaliland or dominica or dominican republic or ecuador or egypt or united arab republic or el salvador or equatorial guinea or spanish guinea or eritrea or estonia or eswatini or swaziland or ethiopia or fiji or gabon or gabonese republic or gambia or "georgia (republic)" or georgian or ghana or gold coast or gibraltar or greece or grenada or guam or guatemala or guinea or guinea bissau or guyana or british guiana or haiti or hispaniola or honduras or hungary or india or indonesia or timor or iran or iraq or isle of man or jamaica or jordan or kazakhstan or kazakh or kenya or "democratic people's republic of korea" or republic of korea or north korea or south korea or korea or kosovo or kyrgyzstan or kirghizia or kirgizstan or kyrgyz republic or kirghiz or laos or lao pdr or "lao people's democratic republic" or latvia or lebanon or lebanese republic or lesotho or basutoland or liberia or libya or libyan arab jamahiriya or lithuania or macau or macao or republic of north macedonia or macedonia or madagascar or malagasy republic or malawi or nyasaland or malaysia or malay federation or malaya federation or maldives or indian ocean islands or indian ocean or mali or malta or micronesia or federated states of micronesia or kiribati or marshall islands or nauru or northern mariana islands or palau or tuvalu or mauritania or mauritius or mexico or moldova or moldovian or mongolia or montenegro or morocco or ifni or mozambique or portuguese east africa or myanmar or burma or namibia or nepal or netherlands antilles or nicaragua or niger or nigeria or oman or muscat or pakistan or panama or papua new guinea or new guinea or paraguay or peru or philippines or philipines or phillipines or phillippines or poland or "polish people's republic" or portugal or portuguese republic or puerto rico or romania or russia or russian federation or ussr or soviet union or union of soviet socialist republics or rwanda or ruanda or samoa or pacific islands or polynesia or samoan islands or navigator island or navigator islands or "sao tome and principe" or saudi arabia or senegal or serbia or seychelles or sierra leone or slovakia or slovak republic or slovenia or melanesia or solomon island or solomon islands or norfolk island or norfolk islands or somalia or south africa or south sudan or sri lanka or ceylon or "saint kitts and nevis" or "st. kitts and nevis" or saint lucia or "st. lucia" or "saint vincent and the grenadines" or saint vincent or "st. vincent" or grenadines or sudan or suriname or surinam or dutch guiana or netherlands guiana or syria or syrian arab republic or tajikistan or tadjikistan or tadzhikistan or tadzhik or tanzania or tanganyika or thailand or siam or timor leste or east timor or togo or togolese republic or tonga or "trinidad and tobago" or trinidad or tobago or tunisia or turkey or turkmenistan or turkmen or uganda or ukraine or uruguay or uzbekistan or uzbek or vanuatu or new hebrides or venezuela or vietnam or viet nam or middle east or west bank or gaza or palestine or yemen or yugoslavia or zambia or zimbabwe or northern rhodesia or global south or africa south of the sahara or sub-saharan africa or subsaharan africa or africa, central or central africa or africa, northern or north africa or northern africa or magreb or maghrib or sahara or africa, southern or southern africa or africa, eastern or east africa or eastern africa or africa, western or west africa or western africa or west indies or indian ocean islands or caribbean or central america or latin america or "south and central america" or south america or asia, central or central asia or asia, northern or north asia or northern asia or asia, southeastern or southeastern asia or south eastern asia or southeast asia or south east asia or asia, western or western asia or europe, eastern or east europe or eastern europe or developing country or developing countries or developing nation? or developing population? or developing world or less developed countr* or less developed nation? or less developed population? or less developed world or lesser developed countr* or lesser developed nation? or lesser developed population? or lesser developed world or under developed countr* or under developed nation? or under developed population? or under developed world or underdeveloped countr* or underdeveloped nation? or underdeveloped population? or underdeveloped world or middle income countr* or middle income nation? or middle income population? or low income countr* or low income nation? or low income population? or lower income countr* or lower income nation? or lower income population? or underserved countr* or underserved nation? or underserved population? or underserved world or under served countr* or under served nation? or under served population? or under served world or deprived countr* or deprived nation? or deprived population? or deprived world or poor countr* or poor nation? or poor population? or poor world or poorer countr* or poorer nation? or poorer population? or poorer world or developing econom* or less developed econom* or lesser developed econom* or under developed econom* or underdeveloped econom* or middle income econom* or low income econom* or lower income econom* or low gdp or low gnp or low gross domestic or low gross national or lower gdp or lower gnp or lower gross domestic or lower gross national or lmic or lmics or third world or lami countr* or transitional countr* or emerging econom* or emerging nation?).ti,ab,sh,kf. 2520890

9 7 or 8 2621245

10 (Trauma adj3 (Surgery or operat*)).ti,ab,kw,kf. 16477

11 exp Surgical procedures, operative/ and trauma.ti,ab. 60684

12 10 or 11 71330

13 exp benchmarking/ 18923

14 exp Population surveillance/ 74781

15 (volume* or benchmark* or rate).ti,ab,kw,kf. 3397899

16 sn.fs. 1043984

17 exp "Health Services Needs and Demand"/ 62792

18 13 or 14 or 15 or 16 or 17 4363207

19 9 and 12 and 18 1642

20 Benchmarking Global Trauma Care Defining the Unmet Need for Trauma Surgery in Ghana.m_titl. 1

21 19 and 20 1

22 (Understanding the health system utilisation and reasons for avoidable mortality after fatal injury within a Three-Delays framework in Karonga, Northern Malawi: a retrospective analysis of verbal autopsy data).m_titl. 1

23 19 and 22 0

24 exp "Wounds and Injuries"/ 1032538

25 (wound* or injury* or injured or injuries or accident* or mortuary).ti,ab,kw,kf. 1366887

26 exp Accidents/ 211853

27 exp Mortuary Practice/ or exp Autopsy/ 46658

28 24 or 25 or 26 or 27 2128650

29 exp "Global Burden of Disease"/ or exp Symptom Burden/ 2404

30 burden*.ti,ab,kw,kf. 351384

31 13 or 14 or 15 or 16 or 17 or 29 or 30 4631316

32 9 and 28 and 31 38060

33 ((avoidable adj5 (injury or mortality)) or (avoid* adj5 fatal*) or (avoid* adj5 death*)).ti,ab,kw,kf. 6091

34 9 and 31 and 33 703

35 22 and 34 1

36 exp Health Services Accessibility/ 137130

37 31 or 36 4717375

38 9 and 37 and 33 726

**Global Health via EBSCOhost search strategy:**

S1 TI(Trauma n3 (Surgery or operat*)) or AB(Trauma n3 (Surgery or operat*)) Expanders - Apply equivalent subjects Search modes - Boolean/Phrase Interface - EBSCOhost Research Databases Search Screen - Advanced Search Database - Global Health 1,125

S2 TI(volume* or benchmark* or rate) or AB(volume* or benchmark* or rate) Expanders - Apply equivalent subjects Search modes - Boolean/Phrase Interface - EBSCOhost Research Databases Search Screen - Advanced Search Database - Global Health 689,174

S3 S1 AND S2 Expanders - Apply equivalent subjects Interface - EBSCOhost Research Databases 308

S4 TI(wound* or injury* or injured or injuries or accident* or mortuary) or AB(wound* or injury* or injured or injuries or accident* or mortuary) Expanders - Apply equivalent subjects Search modes - Boolean/Phrase Interface - EBSCOhost Research Databases Search Screen - Advanced Search Database - Global Health 144,516

S5 TI(burden*) or AB(burden*) Expanders - Apply equivalent subjects Search modes - Boolean/Phrase Interface - EBSCOhost Research Databases Search Screen - Advanced Search Database - Global Health 98,401

S6 S2 OR S5 Expanders - Apply equivalent subjects Search modes - Boolean/Phrase Interface - EBSCOhost Research Databases Search Screen - Advanced Search Database - Global Health 763,357

S7 S4 AND S6 Expanders - Apply equivalent subjects Search modes - Boolean/Phrase Interface - EBSCOhost Research Databases Search Screen - Advanced Search Database - Global Health 32,232

S8 TI(health service* n3 access*) or AB(health service* n3 access*) Expanders - Apply equivalent subjects Search modes - Boolean/Phrase Interface - EBSCOhost Research Databases Search Screen - Advanced Search Database - Global Health 7,122

S9 S7 AND S8 Expanders - Apply equivalent subjects Search modes - Boolean/Phrase Interface - EBSCOhost Research Databases Search Screen - Advanced Search Database - Global Health 46

**Web of Science Search Strategy (v0.1)**

*# Database: SciELO Citation Index*

*# Entitlements:*

*- SCIELO.SCIELO: 2002 to 2024*

*# Searches:*

*1: TS=((trauma near/3 (Surgery or operat*))) Date Run: Wed May 22 2024 14:40:37*

*GMT+0100 (British Summer Time) Results: 389*

*2: TS=((volume* or benchmark* or rate)) Date Run: Wed May 22 2024 14:42:28 GMT+0100*

*(British Summer Time) Results: 92959*

*3: #2 AND #1 Date Run: Wed May 22 2024 14:42:49 GMT+0100 (British Summer*

*Time) Results: 85*

*4: TS=(wound* or injury* or injured or injuries or accident* or mortuary) Date Run: Wed May*

*22 2024 15:05:40 GMT+0100 (British Summer Time) Results: 25614*

*5: TS=(burden*) Date Run: Wed May 22 2024 15:05:57 GMT+0100 (British Summer*

*Time) Results: 4862*

*6: #5 OR #2 Date Run: Wed May 22 2024 17:58:50 GMT+0100 (British Summer*

*Time) Results: 96822*

*7: #6 AND #4 Date Run: Wed May 22 2024 18:01:54 GMT+0100 (British Summer*

*Time) Results: 4217*

*8: TS=(health service* near/3 access*) Date Run: Wed May 22 2024 18:01:59 GMT+0100*

*(British Summer Time) Results: 1870*

*9: #8 AND #7 Date Run: Wed May 22 2024 18:02:07 GMT+0100 (British Summer*

*Time) Results: 5*

**Globus Index Medicus Search Strategy:**

(trauma) AND (volume* or benchmark*) AND (surger* or operat* or procedur*) - 944

(volume* OR burden* OR benchmark* OR rate*) AND (wound* OR injury* OR injured OR injuries OR accident* OR mortuary) AND (avoidabl*) - 49
